# Systemic Inflammatory Disorders, Immunosuppressive Treatment and Increase Risk of Head and Neck Cancers—A Narrative Review of Potential Physiopathological and Biological Mechanisms

**DOI:** 10.3390/cells12172192

**Published:** 2023-09-01

**Authors:** Nuno Vale, Mariana Pereira, Rui Amaral Mendes

**Affiliations:** 1 OncoPharma Research Group, Center for Health Technology and Services Research (CINTESIS), Rua Doutor Plácido da Costa, 4200-450 Porto, Portugal; mariana.m.pereira2097@gmail.com; 2 CINTESIS@RISE, Faculty of Medicine, University of Porto, Alameda Professor Hernâni Monteiro, 4200-319 Porto, Portugal; 3 Department of Community Medicine, Information and Health Decision Sciences (MEDCIDS), Faculty of Medicine, University of Porto, Rua Doutor Plácido da Costa, 4200-450 Porto, Portugal; 4 Department of Oral and Maxillofacial Medicine and Diagnostic Sciences, Case Western Reserve University, 10900 Euclid Ave, Cleveland, OH 44106-7401, USA

**Keywords:** head and neck cancers, immunosuppression, systemic inflammatory diseases

## Abstract

Head and neck cancers (HNCs) are known to present multiple factors likely to influence their development. This review aims to provide a comprehensive overview of the current scientific literature on the interplay between systemic inflammatory disorders, immunosuppressive treatments and their synergistic effect on HNC risk. Both cell-mediated and humoral-mediated systemic inflammatory disorders involve dysregulated immune responses and chronic inflammation and these inflammatory conditions have been associated with an increased risk of HNC development, primarily in the head and neck region. Likewise, the interaction between systemic inflammatory disorders and immunosuppressive treatments appears to amplify the risk of HNC development, as chronic inflammation fosters a tumor-promoting microenvironment, while immunosuppressive therapies further compromise immune surveillance and anti-tumor immune responses. Understanding the molecular and cellular mechanisms underlying this interaction is crucial for developing targeted prevention strategies and therapeutic interventions. Additionally, the emerging field of immunotherapy provides potential avenues for managing HNCs associated with systemic inflammatory disorders, but further research is needed to determine its efficacy and safety in this specific context. Future studies are warranted to elucidate the underlying mechanisms and optimize preventive strategies and therapeutic interventions.

## 1. Introduction

**Figure 1:**
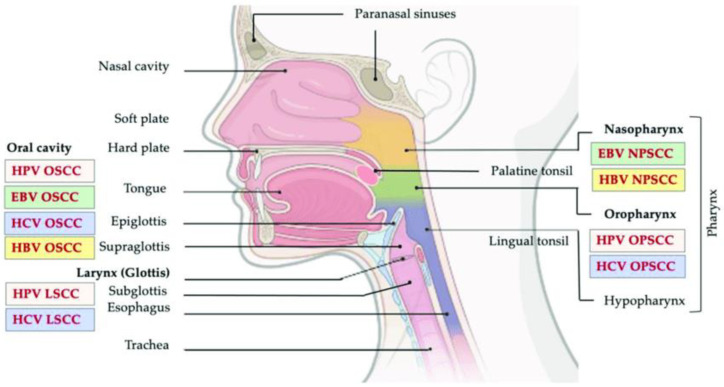
Head and neck cancer regions. This image shows the relation between the different anatomical regions of the head and neck and viral-associated cancers. Adapted from [2].

Head and neck cancers (HNC) account for 3% of all cancers in the United States, with a fourth of the patients succumbing to the disease every year [1]. There are several classical risk factors associated with HNCs, such as alcohol use, smoking, human papillomavirus (HPV) and Epstein–Barr virus (EBV) infections—and more general risk factors include chronic inflammation, and immunodeficiency (Figure 1).

An association between chronic systemic inflammation and risk of malignancy has been well established [3,4,5]. Poor oral hygiene and consequent inflammatory periodontal disease may predispose to oral cancers [6,7,8,9]. Interestingly, periodontitis may increase the risk of non-oral cancers such as pancreatic [5] and breast cancers [10]. These observations are likely attributable to increased levels of products of chronic inflammation including known tumor promotors such as interleukin (IL)-6 (IL-6), tumor necrosis factor alpha (TNF-α), IL-12, IL-23, IL-1β and nuclear factor kappa B (NF-κB) as well as transforming growth factor beta (TGF-β) and IL-10 which might inhibit anti-tumor immunity [11,12,13,14].

Likewise, immune deficiency is associated with increased HNC risk. For instance, patients with acquired immunodeficiency syndrome (AIDS) are significantly more likely to develop mucosal head and neck squamous cell carcinomas (SCCs), tend to develop the disease at a younger age and with worse prognosis than human immunodeficiency virus (HIV)-negative patients [15]. Furthermore, it is well known that patients having undergone solid organ transplantation are also at a higher risk of developing HNCs [16].

Moreover, immunosuppression likely predisposes to oncogenesis by suppressing cell-mediated immunity, the primary means by which the immune system eliminates tumor cells. CD4^+^ Th1 cells and CD8^+^ T-cells are generally regarded as tumor-suppressant, majorly through the activity of interferon gamma (IFN-γ) [13]. Stimulated by Tumor-Associated Antigens (TAA) presented by the tumor cells, T-cells are activated to secrete, perforins, granzymes cytokines and IFN-γ, which cause direct cell cytotoxicity [17]. Cytolytic T lymphocytes (CTLs) and natural killer cells (NKCs) are also largely regarded to hinder tumors, including HNCs [18]. However, it must be noted that not all T-cells have anti-tumor activity. CD4^+^CD25^+^Foxp3^+^ T cells, regulatory T-cells (T-regs) promote tumor activity by inhibiting the activation of both CD4^+^ and CD8^+^ cells. T-regs also secrete pro-tumor cytokines such as TGF-β. Regardless, it must be noted that certain emerging evidence contradicts the pro-tumor activities of T-regs, ergo their oncogenic role remains unclear [13]. Another line of immune cells, IL-17 producing CD4^+^ T-cells (Th17), are stimulated and expanded by the expression of TGF-β, IL-6 and IL-23 in the tumor micro-environment, and are believed to be implicated in tumorigenesis. While, similarly to T-regs, Th17 cells are also believed to have a pro-tumor effect, contradictory reports leave their exact roles to be inconclusive until further investigation [13].

Patients suffering from auto-immune diseases often face a dual challenge, linked to the chronic inflammation associated with the disease’s flares, but also with the immunosuppression arising from the disease’s treatments, making it rather important to assess their risk to develop HNCs, considering that both chronic inflammation and immunosuppression may be regarded as potential risk factors associated with development of HNCs.

## 2. Materials and Methods

### 2.1. HNC Incidence in Systemic Inflammatory Diseases

We defined HNCs as oral, pharyngeal, oro-pharyngeal, lip, nasal cavity and laryngeal cancers—and excluded thyroid, skin and esophageal cancers. Numerous NCBI PubMed searches relating selected system inflammatory conditions and associated reports of head and neck cancers were conducted. Relevant reports and reviews in PubMed results were isolated and reviewed.

### 2.2. Immune Pathways Review

NCBI Pubmed was mainly used to gather information about the specific immune pathways involved in different systemic diseases, and their treatments. To obtain a more thorough search of pathways, queries using UpToDate^®^ (Waltham, MA, USA), Web of Science™ (Clarivate Plc, PA, USA) and Google Scholar were also conducted.

## 3. Results

### 3.1. HNCs and the Immune System

The systemic immune-inflammation index (SII) has emerged as a pertinent biomarker for prognostic assessment across different malignancies. A recent meta-analysis demonstrated a noteworthy correlation between elevated pretreatment SII and unfavorable outcomes, including worsened overall survival, disease-free survival, progression-free survival, as well as higher T and N classifications, among patients diagnosed with head and neck cancer (HNC) [19]. Likewise, another recent systematic review reported an incidence of oral carcinoma in immunosuppressed patients of 200 new cases per 100,000 [20].

Overall, the increased incidence of head and neck cancer in patients with systemic inflammatory diseases can be attributed to a combination of factors, including chronic inflammation, genetic predisposition and epigenetic changes, tissue damage, interactions with viral infections and altered immune responses associated, or not, with immunosuppressive treatments. This complex interplay creates an environment that may favour the initiation and/or progression of cancerous cells within the head and neck region [20].

In fact, inflammation is known to contribute to DNA damage, cell proliferation, angiogenesis and evasion of immune surveillance, which can later promote tumorogenesis. Similarly, altered immune response in systemic inflammatory diseases can lead to dysregulated immune responses, an aspect often impacted by the immunosuppressive treatments usually associated with systemic inflammatory diseases, which weaken the immune system’s ability to identify and eliminate abnormal cells, including cancer cells. In fact, this imbalance can create an environment where the immune system may not effectively target and eliminate early cancerous cells, allowing them to progress into full-blown tumors [20].

One must also consider the likely detrimental impact of carcinogenic cytokines, as pro-inflammatory cytokines may act as signaling molecules which then stimulate cell growth, survival and angiogenesis, fostering an environment conducive to cancer development.

Overall, iinflammatory diseases can be broadly categorized as cell-mediated or humoral, based on the immune mechanisms involved in their pathogenesis. When comparing the risk for head and neck cancer associated with these inflammatory diseases, as well as their response to immunotherapy treatments, there are some notable differences.

Cell-Mediated Inflammatory Diseases:

Conditions such as oral lichen planus, oral submucous fibrosis and chronic graft-versus-host disease (GVHD) are characterized by cell-mediated immune responses, which is an adaptative immunity response that is based on T-cells. These cells are physically present and actively recognize the antigen and attack the target, which is a slower process [21]. These diseases are generally associated with a higher risk of developing HNC, as the chronic inflammation and tissue damage caused by these diseases can create a favorable environment for the development of malignant changes [22]. GVHD especially, which is related to hematopoietic stem cell transplantation, has been associated with the development of malignancies, namely oral carcinoma [23,24,25,26,27]. Since manifestations of oral squamous cell carcinoma (OSCC) can emerge within a relatively brief latent period, even as early as one year following hematological stem cell transplant (HSCT), it becomes plausible that the activation of the PI3K pathway initiates during the early stages of graft-versus-host disease (GvHD) development (16). The identification of a connection between AKT signaling, which constitutes a downstream element of the PI3K pathway, and the B-cell activity associated with GvHD further strengthens the implication of this pathway in the genesis of GvHD [28]. Moreover, HNC can appear in GVHD patients due to the combination of immunosuppressants used to treat it and radiotherapy, and the oral cancers are normally of squamous nature [29]. Immunotherapy treatments, particularly immune checkpoint inhibitors such as pembrolizumab and nivolumab, have shown promising results in the treatment of HNC associated with cell-mediated inflammatory diseases [30]. These drugs help unleash the body’s immune system to target and destroy cancer cells. However, the response to immunotherapy can vary among individuals, and not all patients may benefit equally [31].

Humoral Inflammatory Diseases:

Diseases such as Sjögren’s syndrome and systemic lupus erythematosus (SLE) involve humoral immune responses [32,33]. These responses are characterized by the production of antigen-specific antibodies by lymphocytes B, a response that is developed quickly [34]. The inflammatory diseases are not as strongly linked to an increased risk of HNC compared to cell-mediated diseases. However, chronic inflammation and immunological dysregulation may contribute to a slightly elevated risk as we will address in more detail. It is also important to highlight that, contrary to what we can observe in cell-mediated inflammatory diseases, the effectiveness of immunotherapy in treating HNC associated with humoral inflammatory diseases is still not well established [35]. Since these diseases involve complex immune system abnormalities, their interaction with immunotherapy agents may differ from that of cell-mediated diseases. Further research is needed to determine the potential benefits of immunotherapy in this context.

Either way, in this paper we present some examples of the relationship between different autoimmune inflammatory diseases and head and neck cancer, as we also highlight that multifactorial, and individual variations can exist. Hence, regular monitoring, early detection and tailored treatment plans are crucial for managing patients with these conditions and mitigating the risk of cancer development.

#### 3.1.1. Incidence of HNCs in Systemic Sclerosis

While there are contradictory findings, some studies report a strong increased risk of HNCs in systemic sclerosis (SSc) patients. In a cohort of 769 SSc patients in an American institution, Derk et al. reported nine patients with diffuse SSc who developed oral and pharyngeal carcinomas, six of which were SCCs of the tongue. One patient had both pharyngeal and tongue SCC within a 4-year period, while another patient developed three separate tongue SCCs. Altogether, the cohort has a standardized incidence ratio (SIR) of 25 for developing tongue carcinoma, and SIR of 9.63 for oropharyngeal carcinomas [36]. In a review of 2053 Taiwanese patients with SSc, Kuo et al. reports an oral cavity and pharyngeal cancer SIR of 3.67 compared to the general Taiwanese population. This value was obtained by dividing the number of cases of oral cavity and pharyngeal cancers by the expected number among the general Taiwanese population, and the data were collected from the National Death Registry in Taiwan and the Taiwan National Health Insurance Research Dataset (NHIRD) [37].

#### 3.1.2. Incidence of HNCs in Inflammatory Bowel Diseases

Inflammatory bowel diseases (IBD) refers to two autoimmune diseases: ulcerative colitis (UC) and Crohn’s disease (CD). While there are a considerable number of case reports of HNCs arising in patients with IBD, no clear incidence ratios have been reported [38,39].

Related case reports include the development of oral SCC in a CD patient who was treated with azathioprine (AZA) for 9 years [40], neck cavity SCC in a CD patient who was successfully treated with infliximab [41], and tongue SCC in a patient being treated with AZA [42].

A 2015 review of cancerous and precancerous lesions in IBD patients provides an excellent review of many related cohort studies [38], and the incidence of HNCs in IBD patients.

#### 3.1.3. Incidence of HNCs in Systemic Lupus Erythematous

The incidence of HNCs in patients with systemic lupus erythematous (SLE) is more strongly associated. In a study of 8751 patients with SLE, Chang et al. reported a 2.16 higher incidence risk ratio of HNCs in patients compared to controls, with the highest incidence being oral cavity cancer (5 of 11 cases). Of note, SLE comorbidity did not affect survival of patients diagnosed with HNCs [43]. A large meta-analysis of 18 studies including a total of 86,069 SLE patients reported the risk of HNCs, not including thyroid cancer, to be 2.31 fold higher [44]. Furthermore, a meta-analysis of 16 studies including 59,662 SLE patients found that SLE patients have a pooled relative risk of 4.19 for laryngeal cancer and 1.54 for cancer of the oropharynx [45]. Another study of 11,763 Taiwanese patients with SLE reported a nasopharyngeal cancer SIR of 4.18, oropharyngeal and laryngeal SIR of 2.02 relative to the general population [46]. Lastly, in 2021, a cohort of 17,854 Korean patients with SLE showed an increased risk of HNC with an SIR of 2.7, the highest SIR among solid malignancies in this study [47].

Albeit only a few, case reports were also found. These include the case reports of SCC of the palate developing in a SLE patient being treated with prednisone and cyclophosphamide (CYC) [48], and tongue carcinoma developing in a SLE patient during pregnancy, five years after heavy treatment with CYC and mesna [49].

#### 3.1.4. Incidence of HNCs in Rheumatoid Arthritis

While there are case reports, no large review or meta-analysis was found regarding the incidence of HNCs in rheumatoid arthritis (RA) patients. Beattie et al. report oral SCC developing in a RA patient being treated with adalimumab [50]. Chainani-Wu et al. also report a case of oropharyngeal SCC developing in a RA patient being treated with both methotrexate (MTX) and etanercept [51].

A review by Philips et al. of 180 patients with HNCs, reports that the use of TNF inhibitors (TNFi) with both RA and HNC did not affect the risk of HNC recurrence or death. This study, however, did not focus on the occurrence of HNC in RA patients being treated with TNFi [52].

#### 3.1.5. Incidence of HNCs in Dermatomyositis

There are sparse case reports of dermatomyositis (DM) patients developing oral and pharyngeal cancer. These include tongue SCC developing in a young patient with DM being treated with AZA and prednisolone [53], tonsillar SCC developing in a 63 year old male patient with DM being treated with prednisone, MTX and hydroxychloroquine for a year (authors view the DM as a paraneoplastic syndrome) [54], tonsillar carcinoma developing in a Caucasian patient with DM after seven months of treatment with AZA and prednisone [55], pleomorphic adenoma with malignant features developing in a 71 year old Caucasian patient with DM [56], and development of tonsillar SCC in two Korean patients with DM, one after a year of treatment with AZA and prednisolone, and the other after two years of treatment with ‘steroids’ [57]. In a report of 136 hospitalized DM patients, one patient developed hypopharyngeal carcinoma, while 4 developed nasopharyngeal carcinoma (NPC) [58].

There is a strong association of DM patients developing NPC, which seems to be the most prevalent in patients of Eastern Asian. NPC was reported to develop in 12/90 DM patients in a hospital in China [59], 12/104 DM patients in a hospital in Taiwan [60], 5/130 DM patients in hospital in Tunisia [61] and 7/68 DM patients in a multiracial cohort of 68 Asian patients [62]. A review of 1154 NPC cases based in a Hong Kong hospital found 10 patients with concurrent DM diagnosis, 9 of which were made prior to NPC diagnosis [63]. The pathogenesis of NPC may be multifactorial in addition to systemic inflammatory illnesses and its immunosuppressive treatment, as EBV has been significantly associated in DM and SLE patients who developed NPC [64]. Incidence of NPC’s developing in DM patients has also been reported in an Israeli ‘white’ Jewish patients being treated with prednisone and MTX [65].

#### 3.1.6. Incidence of HNCs in Psoriasis

Several studies have explored the incidence of oral, pharyngeal and laryngeal cancer in psoriasis patients. A cohort study of 9773 psoriasis patients in Sweden found SIRs of 2.8 for oral and pharyngeal cancers, and 1.43 for laryngeal cancer [66]. A different cohort study of 6905 psoriasis patients found a SIRs of 1.7 for oral cancer, 2.9 for pharyngeal cancer, and 2.0 for laryngeal cancer. Men had higher risk than females, with SIRs of 2.1 for oral cancer, 4.1 for pharyngeal cancer and 2.4 for laryngeal cancer [67]. Furthermore, a study of 5687 hospitalized patients with psoriasis in Finland reports SIRs of 0.7 for oral cancer, 1.3 for pharyngeal cancer and 2.9 for laryngeal carcinoma [68].

Case reports of HNC occurrence in psoriasis patients can also be found in the literature. For example, Seoane et al. report a case report of a gingival mucinous adenocarcinoma of a minor salivary gland in a patient with a history of psoriasis and chronic gingivitis [69], Carlesimo et al. report two cases of salivary gland tumors developing in two psoriatic patients being treated with TNFi [70], and Yamamoto et al. report the development of oral adenocarcinoma in a psoriasis patient receiving short-term cyclosporine (CsA) therapy [71].

### 3.2. Pro-Tumor Cytokines and Inflammatory Signaling in Queried Auto-Immune Systemic Diseases

#### 3.2.1. Systemic Sclerosis

Serum and skin presence of TGF-β (cancer-promoting) seems to take place early in pathogenesis of both local and diffuse SSc [72]. Serum IL-6 and C-reactive protein (CRP) (cancer-promoting) are also elevated in SSc and correlate with clinical disease activity [73].

In 54 SSc patients, there were significantly increased serum levels of IL-6, TNF-α, monocyte chemoattractant protein 1 (MCP1) (cancer-promoting), but also reduced IFN-γ and macrophage derived chemokine (MDC) (cancer-suppressive). Peripheral blood mononuclear cells supernatant also showed increased levels of IL-6, IL-8, IL-10, IL-18, macrophage inflammatory protein 1 gamma (MIP-1γ) (cancer-promoting) and TNF-α (cancer-suppressive). Levels of IL-6, IL-10 and MDC could discriminate between limited and diffuse forms of the disease [74]. Increased levels of circulating IL-1 (cancer-promoting), IL-2, IL-2 receptors (IL-2R) (cancer-suppressive), IL-4, IL-8, IL-17 (cancer-promoting) and TNF-α have been reported in patients with SSc [75,76,77,78].

Serum elevations of vascular cell adhesion molecule 1 (VCAM-1) and E-Selectin (cancer-promoting) were noted in SSc and were associated with disease severity [79]. The chemokine platelet activating factor 4 (CXCL4) (cancer-promoting), a signal for chemotaxis, is also increased in patient plasma levels and correlates with disease complications and progression [80]. CXCL4 may also have roles in inhibiting angiogenesis [81].

#### 3.2.2. Systemic Lupus Erythematous

Unlike in other auto-immune diseases, most SLE patients have significant serum acid-labile IFN-α (cancer-suppressive) levels [82], which correlates with disease flares and remission [83]. While one study found that about a quarter of SLE patients have higher levels of TNF-α relative to controls [84], other studies suggest decreased production of TNF-α in a subpopulation of SLE [85,86].

In about half of SLE patients with active disease, serum IL-2 is also increased. This may be normalized with immunosuppressive therapy [87,88]. Serum IL-2R is also elevated in about a third of the patients and it correlates with disease activity and the Westergren erythrocyte sedimentation rate (ESR) [89,90]. Lymphocytes production of activated TGF-β is markedly decreased in active diseases, and less profoundly decreased in treated SLE [91].

Lastly, animal models, specifically NZB/NZW mice who develop an SLE-like illness, also suggest decreased production of TNF-α [92]. Mice with higher production of TNF-α are more resistant to the development of the disease [93].

The role of cytokine expression patterns in SLE triggering HNC is not fully understood and is likely multifactorial. In fact, the cytokine imbalances in SLE can lead to a breakdown of self-tolerance, where the immune system targets normal cells, with IFN-gamma and TNF-alpha contributing to tissue damage, cellular stress and inflammation in various organs. Moreover, immune surveillance, which normally identifies and eliminates abnormal cells, may be compromised in SLE Hence, tissue damage and associated DNA mutations are likely to go undetected, since SLE patients often experience immune dysfunction and suppression, thus weakening the immune system’s ability to detect and eliminate cancerous cells that may arise due to abovementioned DNA mutations.

#### 3.2.3. Rheumatic Arthritis

Serum and synovial TNF-α is a central cytokine in the pathogenesis of RA, which has numerous effects including antigen-presenting cells production of granulocyte macrophage colony stimulating factor (GM-CSF) (cancer-promoting), proliferation of T-cells [94] and possible disruption of regulatory T-cell suppressive effects [95]. Macrophage-produced increases in IL-15, which increase TNF-α production in peripheral blood T cells, is one-local-source of the cytokine [96]. GM-CSF in synovial tissue supernatant, produced by synovial macrophages, may initiate T-cell activation in response to specific antigens in an RA patient [97] and blocking it using a monoclonal antibody reduces disease severity [98].

Lastly, IL-20 (cancer-promoting), a pro-inflammatory cytokine that promotes oral tumor growth, migration and tumor-associated inflammation [99], is also increased in plasma of RA patients [100].

#### 3.2.4. Inflammatory Bowel Diseases

**Figure 2:**
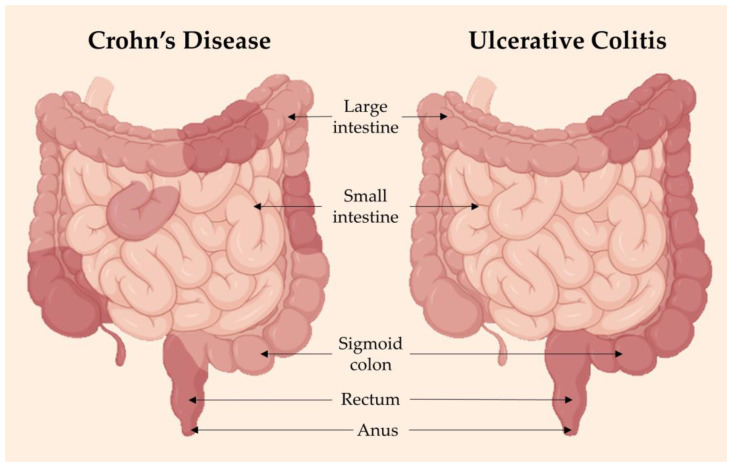
Inflammatory bowel disease (IBD) is a term referring to two conditions: Crohn’s disease (CD) and ulcerative colitis (UC), both of which are characterized by chronic inflammation and damage of the gastrointestinal tract. CD (left) has inflammation in patches throughout the large bowel, while UC has uniform and continuous inflammation throughout. If not managed well, IBD flare-ups can be aggressive and debilitating to a person’s quality of life. Adapted from [102]. Image made using BioRender software (2023).

Although primary symptoms of inflammatory bowel diseases (IBD) concern the intestines, extra-intestinal manifestations such as arthralgia, arthritis and cachexia are also present, inferring possible systemic deregulation of inflammatory mediators [101]. Crohn’s disease (CD) can develop at any point of the gastrointestinal tract, more commonly in the end of the small intestine, causing inflammation in all layers in random areas, even if they are adjacent to healthy parts of the intestines. Meanwhile, ulcerative colitis (UC) is confined to the colon and rectum, causing inflammation in continuous areas and only in the inner lining of these areas (Figure 2). Complications associated with CD are anal fissures, strictures, fistulas, bowel obstruction, malabsorption and colon cancer. In UC, the complications include severe dehydration, colon perforation, toxic megacolon and colon cancer [102].

However, the underlying pathogenesis of manifestations may be different when comparing UC and CD— whereas CD seems to be primarily a Th1 cytokine mediated disease, with overproduction of IFN-γ, UC seems to be a “Th2-like” mediated disease with overproduction of IL-5 and IL-13 (cancer-promoting), but with normal IFN-γ and IL-4 levels. Furthermore, while involvement of a Th17 response is strongly suggested in CD, this is a drastically smaller source of effector cytokines than the Th1 response [103].

While serum studies of IBD have resulted in contradictory results, significant inflammatory markers have been recognized. For example, one study found increased serum levels of IL-6 and IL-7 (cancer-suppressive) in CD, and increased TNF-α, eotaxin and growth-related oncogene (a chemokine) in UC patients. Both CD and UC also demonstrated increased IL-8 relative to the controls [104]. A review of the studies using R&D Systems enzyme-linked immunosorbent assay (ELISA) suggests that IL-6 seems to be increased in both UC and CD relative to controls. However, testing using other technology is still contradictory [104].

Lastly, sera cytotoxic CD13 antibodies have been reported in 66% of human cytomegalovirus-immunoglobulin-G (HCMV-IgG) positive UC patients and 58% of HCMV-IgG positive CD patients [105]. In a study of 50 CD patients in Turkey, they had elevated ESR and CRP at baseline compared with healthy controls; however, no differences in Th17 levels were found relative to controls or disease activity [106].

#### 3.2.5. Psoriasis

Systemic manifestations of psoriasis range from cardiovascular disease to malignancy, diabetes, IBD and other autoimmune disorders [107,108,109,110,111].

Although there have been multiple studies on the systemic inflammatory markers found in psoriasis patients, different studies report conflicting findings. However, a review of 78 studies found that standardized mean differences (SMD) of IL-6, CRP, TNF-α, E-selectin and intracellular adhesion molecule 1 (ICAM-1) were modestly but significantly higher in patients with severe disease relative to controls. Interestingly, there was no difference in the pooled SMD of serum IL-1β or IL-10 when comparing psoriasis patients to controls [112].

#### 3.2.6. Dermatomyositis

Serum antibodies both favoring and negating the development of malignancy have been found in DM. For instance, antibodies to transcriptional intermediary factor 1 gamma (anti-p155 and anti-p155/140) [113,114], and antibodies to the nuclear matrix protein (anti-MJ or anti-p140) [115,116] were noted in DM patients.

There also seems to be elevations of TNF, IL-1 and IL-6 in both DM and polymyositis (PM) patients [117]. A study of 56 patients with juvenile DM not only correlated serum IFN-signature with DM, but also serum IL-6 [118]. Serum IL-15 (cancer-suppressive), IL-17, B-cell activating factor were found to be elevated in DM relative to healthy patients. Serum IL-18 was found to not only be elevated, but also correlate with disease activity in DM [119].

In one study, the number of anti-inflammatory T-reg cell population, and concentrations of serum TGF-β and IL-10 were also significantly lower in DM patient compared to healthy patients [120]. However, some studies have rather shown an increase in the serum IL-10 in patients with DM [119]. A different study also found significantly elevated levels of serum IL-2R and TNF-R1 in patients with DM [121].

Table 1 is a summarized view of all the above-mentioned cytokines, chemokines and other immune/inflammatory response molecules differently expressed in the diseases studied in this paper, in comparison with those differently expressed in HNC.

### 3.3. Anti-Tumor Cell-Mediated Immunity Pathways in Queried Auto-Immune Systemic Diseases

#### 3.3.1. Rheumatic Arthritis

There may be an imbalance towards increased Th1 and Th17 lymphocytes, and decreased Th2 and regulatory T-cells in patients with RA [122]. However, a study of 107 patients with RA found that 20% were anergic as demonstrated by skin tests [123]. A different study of 14 patients also found depressed cell mediated immunity, specifically delayed hypersensitivity reaction [124]. However, both studies report therapy as a possible source of depressed cell mediated immunity, with the latter study finding an association with use of prednisolone and AZA [124].

A series of studies suggest that while NKCs are increased in the synovial fluid of RA patients, they are found in lower numbers and express lower activity in blood [125,126].

#### 3.3.2. Systemic Sclerosis

A study of 35 SSc patients found upregulation of intracellular IFN-γ and TGF-β (and increased levels of circulating IL-17, IL-6, IL-23 and IL-1α) [127]. Studies of patients with SSc have found reduced antibody-dependent cell mediated cytotoxicity and NKC activity, specially early in the illness of the diffuse-type SSc [128].

Another study of 28 patients with SSc found impaired lymphocyte transformation response relative to controls [129].

#### 3.3.3. Systemic Lupus Erythematous

Animal models, specifically, NZB/NZW mice who develop an SLE-like illness, also suggest increased production of IFN-γ, and those treated with monoclonal antibody to IFN-γ show decreased mortality [92].

One study reported reduced antibody-dependent cell mediated toxicity by peripheral mononuclear cells from patients with SLE [128].

A different study of nine patients also found depressed cell mediated immunity, specifically delayed hypersensitivity reaction [124].

Lastly, a series of studies suggest that patients with SLE have lower number of NKCs, and with decreased activity and lower perforin [130].

#### 3.3.4. Inflammatory Bowel Disease

Unlike UC, CD seems to be primarily a Th1 cytokine mediated disease, with overproduction of IFN-γ [103]. In fact, increased serum levels of IFN-γ have been reported [104]. In a study of 54 IBD patients, authors report increased intracellular IL-2 and IFN-γ in both resting and activated CD4^+^ and CD8^+^ cells relative to healthy controls [131].

In a study of 30 patients with CD, decreased cell mediated cytotoxicity was noted relative to healthy controls, and the decrease was weakly associated with disease activity [132]. Furthermore, a different study found significantly decreased circulating NKC activity in CD patients relative to normal controls. The extent of this decrease correlated with disease activity. No such depression was observed in UC patients [133].

#### 3.3.5. Psoriasis

A study found reduced antibody-dependent cell mediated cytotoxicity in patients with psoriasis compared to controls [128]. Decreases in cell-mediated immunity have been reported in other studies. For example, one review reports that while no significant differences in lymphocyte subpopulation frequency were found in psoriasis patients, significant decreases in B-cell response, T-helper function, polymorphonuclear neutrophils (PMN) chemotaxis, PMN and monocyte mediated phagocytosis and lymphokine release were found [134].

While studies suggest increased NKCs in psoriatic plaques, there also seems to be decreased NK and NK-T cells in peripheral blood of psoriatic patients [135,136]. One study even reports decreased capacity of peripheral NKCs (from 45 psoriatic patients) to kill a tumor cell line [137]. A series of studies suggest that peripheral NK-T cells of patients with psoriasis are decreased. This decrease diminishes in response to therapy; however, peripheral NK-T cells do not return to the same level as in normal patients. The reason for this decrease is unclear [136].

Despite the supporting mounting evidence, this decrease in peripheral NKCs in psoriasis patients is still under debate as a series of studies do not report similar findings [138].

#### 3.3.6. Dermatomyositis

Although IFN-γ itself is not detected in high levels in the blood of DM patients, IFN-γ signature (IFN-inducible proteins and transcripts) are highly upregulated [139]. In fact, another study found that the upregulation of IFN-inducible gene expression correlated with disease activity in DM or PM [140]. However, this association with disease activity may be confounded by the presence of specific autoantibodies [119]. Moreover, a study of 50 PM patients and 49 DM patients found a decreased percentage of CD8^+^ lymphocytes, and decreased IFN-γ expression of CD4^+^ and CD8^+^ cells. Similar changes were not noted in PM [141].

It should be noted that the patho-immunology of DM and PM are different in that DM presents abnormal cellular immunity while PM presents with abnormal humoral immunity.

DM seems to be a disease of decreased cellular immunity. Similar to other rheumatic diseases, patients with DM seem to have decreased CD8^+^ T-cells and NKCs in their peripheral blood [142,143,144]. Other evidence suggests that while DM patients have a depression of circulating lymphocyte counts, there is also a simultaneous increase in B-cell count/humoral immunity activity, suggesting a humoral pathophysiology [145,146]. However, decreased peripheral blood CD4^+^ and CD8^+^ cells do correlate with disease activity/therapy, and risk of death in DM patients [147]. Lastly, the mean absolute peripheral blood lymphocyte count in patients with paraneoplastic DM does not seem to statistically differ from DM patients without malignancy [144].

### 3.4. Effects of Anti-Biological and Immunosuppressive Drugs on Cell Mediated Immunity

#### 3.4.1. Glucocorticoids

By inactivating key transcription factors (e.g., NF-kB, and activator protein 1), upregulating cytokine inhibitory proteins (e.g., I kappa B), and speeding degradation of cytokine mRNAs, glucocorticoids (GCS) inhibit the production of numerous cytokines, including IL-1β, TNF-α, IL-2, IL-3, IL-4, IL-5, IL-6, IL-8, IL-10, IL-12 and IFN-γ [148].

By interfering with expression of adhesion molecules, GCS also impair ability of leukocytes to locate to the site of inflammation, leading to defective cellular immune response [149].

Despite the strong cellular response to GCS, it seems that treatment shifts patients’ cellular activity towards to Th2 profile while inhibiting Th1 profile. While some T-cell types, such as CD4^+^ and CD8^+^, undergo apoptosis in presence of GCS, others, such as NKCs, do not [150]. It seems that activated CD4^+^ Th1 cells are more susceptible to GCS induced apoptosis [151]. In fact, it seems that GCS shifts patients’ cytokine profile from Th1 to Th2 cytokines by more markedly inhibiting Th1 cytokine secretion [152]. This preference towards Th2 cytokine secretion may be resulting from GCS’ increased inhibition of IL-12, relative to IL-10 [153,154].

This preference may also promote selective differentiation of native T-cells to Th17 T-cells [155]. Specific Th17 subsets, specifically those expressing multi-drug resistance type 1 may actually be resistant to suppressive effects of GCS [156].

#### 3.4.2. Azathioprine

**Figure 3:**
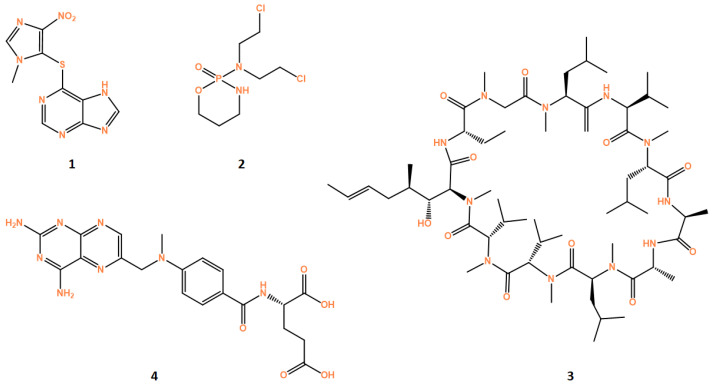
Structures of drugs azathioprine (**1**), cyclosporine (**2**), cyclophosphamide (**3**) and methotrexate (**4**). All structures were obtained using ChemDraw software (version 12.0, PerkinElmer, Inc., Waltham, MA, USA).

Azathioprine (AZA, **1**, Figure 3) is a purine analog derivative of 6-mercaptopurine that competitively inhibits enzymes of de novo purine base synthesis. It is included in the disease-modifying antirheumatic drugs (DMARD) used in refractory patients.

AZA can selectively reduce peripheral NKCs [157]. It has also been described to lead to a dose-dependent inhibition of T-cell proliferation [158]. It also has been noted to induce T-cell apoptosis in CD patients by blocking Ras-related C3 botulinum toxin substrate 1 (Rac1) and its target genes, such as mitogen-activated protein kinase, NF-kB and B-cell lymphoma-extra-large. This suppression leads to a mitochondrial pathway of apoptosis [159].

One study showed that CD patients who respond to AZA have significantly higher peripheral T-cell apoptosis and lower IFN-γ expression relative to non-responders [160].

AZA does not appear to reduce serum levels of IL-6 or soluble IL-2R [157]. However, a study using cultured splenic mononuclear cells showed not only inhibited Th1 cytokine production from AZA exposure, but also dose dependent decreases in IFN-γ and TNF-α [161].

AZA treatment decreased numbers of CD4^+^CD69^+^ and CD4^+^IL-17^+^ cells in SLE patients. While systemic autoimmune diseases may already be associated with lymphopenia, AZA induced lymphopenia in SLE patients independent of SLE-associated lymphopenia. Furthermore, AZA-associated lymphopenia in SLE patients resulted in effector T-cells and T-regs with lower proliferation and suppressive activity when compared to that of patients with SLE-associated lymphopenia [162].

AZA-associated lymphopenia has also been reported in IBD patients [163,164].

#### 3.4.3. Cyclophosphamide

Cyclophosphamide (CYC, **2**, Figure 3) is an alkylating agent that forms cross-links with DNA, thereby interfering with DNA replication [165]. The dosage and administration schedule of CYC used to treat patients can cause variable immunological activities, which can be used to treat cancer, auto immune diseases and others. High dose CYC (e.g., doses of 100–200 mg/m^2^ of BSA [166]) are used to treat autoimmune disease, while low doses (1–3 mg/kg) can be used to treat cancer [167].

Evidence regarding use of high dose CYC demonstrates different effects on the immune system, as it is often associated with immunosuppression. CYC administration has been shown to cause increased delayed hypersensitivity skin reactions to specific antigens [168]. Short term administration of 50–250 mg/kg^−2^/day of CYC produces a dose-dependent lymphopenia and neutropenia [169].

However, the use of a single high dose CYC administration has been shown to eradicate tumor in a CD4^+^ T-cell dependent manner [166].

It is evident that high dose CYC (200 mg/kg) severely decreases the number of all T-cell subsets by >90%. It also decreased the ratio of CD4^+^CD25^+^ to CD4^+^ T cells. It seems that while both low dose and high dose CYC may have anti-tumor effects, low dose CYC does so through depletion of CD4^+^CD25^+^ T-cells and, thus, increased anti-tumor effect, while high dose CYC does so through alkylation and direct cytotoxicity [170].

#### 3.4.4. Cyclosporine

Cyclosporine (CsA, **3**, Figure 3) is a calcineurin inhibitor that is used for immunosuppression in many contexts, including refractory RA, severe UC, psoriasis/psoriatic arthritis, DM, SLE and SSc. With the advent of biological agents, the use of CsA has diminished in rheumatic disease [171].

While CsA does not affect already activated T-cells, it can cause a total block of the proliferation of all inactivated T-cells [172]. CsA evidently blocks expression of IL-2, IL-3, IL-4, TNF-α, CD40L, GM-CSF and IFN-γ [172,173,174,175].

It has also been described to lead to a dose dependent inhibition of T-cell proliferation and production of IFN-γ [158]. Furthermore, CsA seems to shift cytokine expression in RA patients from a Th1 to a Th2 profile [176]. However, conflicting evidence is reported, as Rentenaar et al. observed that Th1/Th2 balance in psoriasis patients treated with CsA was not affected [177]. Also in end-stage renal disease patients, CsA seems to increase TGF-β expression [178]. Lastly, studies report CsA inhibition of T-cell cytotoxicity and cytotoxic T-cell lines [179,180].

#### 3.4.5. TNF-α Inhibitors

Several TNFi have been approved to treat numerous rhematic diseases in the Unites States, including etanercept, infliximab, adalimumab, certolizumab and golimumab. While very effective, the risk of malignancy is a significant hypothetical adverse effect of TNFi. This risk has been significantly studied; however, differences in study design, specific cancer type (e.g., lymphoma vs. other), and other challenges have led to contradictory findings [181,182,183,184,185,186,187,188]. While one study found no increase in risk of recurrence of HNCs in patients being treated with TNFi [52], no major study seems to have prospectively studied the risk for initial HNC.

As expected, TNFi increases expression of FoxP3 cells and the suppressive functions of CD4^+^CD25^+^ cells. However, there is question of exactly which subsets of T-regs are increased by TNFi, and whether that is a significant mechanism for immunosuppression [189]. Studies have demonstrated that whether alone or in combination with other rheumatic disease treatments, TNFi therapy decreases IFN-γ, and Th1 cytokine expression [190,191,192,193,194,195]. Decreases in Th17 and increases in T-regs are also seen ensuing TNFi therapy [190,195].

However, the influence of TNFi on immune system is more complicated. While one study found decreases in Th17 cells and IFN-γ producing CD8^+^ T-cells and increases in the proportion of T-regs in response to TNFi therapy, the percentage of IFN-γ-producing NK cells was also increased [190]. Another study found that TNFi therapy in psoriasis and IBD patients led to increased peripheral IFN-γ, IL-17, IL-10 and the proliferative response of CD4^+^ T-cells to T-cell receptor stimulation, while biopsied target tissues showed decreased Th1/Th17 cytokines and inflammatory genes [196]. A different study found that while in some RA patients TNFi therapy was successful and led to decreased Th1, Th17 and IL-21, in other RA patients therapy was unsuccessful and led to expansion of Th1/Th17 lymphocytes, and increased IL-21 [197]. Another study reports different findings specific to TNFi was used: while etanercept and infliximab led to increased Th1 prevalence 4 weeks after beginning therapy in RA patients, adalimumab led to a stable Th1 prevalence [198].

Study of 21 patients with psoriasis showed that adalimumab downregulates Th1, Th17 and Th22 at 12 weeks relative to the baseline of the patients. At baseline, serum levels of effector cytokines (IFN-γ, IL-17 and IL-22) and proinflammatory cytokines (IL-6, TNF-α) were higher in psoriasis patients relative to healthy controls. All serum cytokines declined significantly by week 12 [191].

In a study of 48 patients UC, 44 of whom were treated with infliximab and 4 with adalimumab, and 26 responded to therapy. In therapy responders, serum decreases in IL-6 were seen in responders at 14 weeks, while an increase in IL-13 and no change in serum levels of IL-1β, IL-4, IL-8, IL-10, IL-12p70, IL-17A, TNF-γ and IFN-γ at week 14 relative to baseline. Among non-responders, serum levels of IFN-γ and IL-12p70 were increased at 14 weeks, while serum levels of IL-1β, IL-4, IL-6, IL-8, IL-10, IL-13, IL-17A and TNF did not change relative to baseline [199].

Another study with 47 psoriasis patients, serum levels of IL-6, TNF-α and proinflammatory adipocytokines (leptin, resistin and visfatin) were higher in psoriasis patients and serum adiponectin (an anti-inflammatory cytokine) was lower in psoriasis patients relative to healthy controls. After 24 weeks of treatment with TNFi, TNF-α and IL-6 were decreased, and only leptin was the only adipocytokine that showed statistically significant decrease [200].

#### 3.4.6. Methotrexate

Methotrexate (MTX, **4**, Figure 3) is an analogue of folic acid. One mechanism by which MTX suppresses immunity is by inhibiting dihydrofolate reductase to reduce production of tetrahydrofolate (THF) and de novo production of nucleic acids [201]. While high dose MTX (>1 g/week) is used to treat malignant disease, lower doses (7.5–25 mg/week) may be used to treat rheumatic diseases [202], especially rheumatoid arthritis and psoriasis [203,204].

Several studies suggest that MTX administrations in RA patients favors shift from expression Th1 cytokines to Th2-cytokines, as seen by decreased IFN-γ, IL-12R, CXCR3 and increased IL-10 [205,206,207,208]. MTX also increases production of CD4^+^CD25^+^ T-regs [205].

However, conflicting evidence is reported. Rentenaar et al., report unaffected Th1/Th2 balance in psoriasis patients treated with MTX [177]. A different study, wherein SLE was induced in a murine model, found that MTX restores the Th1 dominant immune state of the disease-induced mice to a Th1/Th2 balance comparable to healthy controls [209].

Lastly, a review of 6806 DMARD treatment courses in RA patients found that the overall risk of cancer was increased in patients being treated with MTX, in comparison to the patients being treated with non-biological DMARDS and TNFi [210].

## 4. Discussion

While some studies report no increased risk of HNCs occurring in patients with systemic inflammatory diseases, many report increased risk, some as high as 25-fold. Despite some existing dispute over the existing data, due to the rarity of HNCs, lack of controls for confounders (e.g., age, sex, alcohol, smoking and betel nut use) in every study or report, and large variability in immunosuppressive treatment regiments make it difficult to arrive on any confident conclusions, significant associations have been reported, and they should be explored in hopes of understanding not only HNCs, but also other devastating cancers of similar etiology in patients suffering from systemic inflammatory diseases.

In fact, a recent systematic review assessed the incidence of oral cancer in patients with systemic immunosuppression and compared it to data from official databases (Globocan, WHO), hence showing an association and possible correlation between immunosuppression and the development of oral cancer. This study not only showed that immunosuppressed patients have a 50 times higher risk of developing oral cancer compared to the general population, but further emphasized the need for establishing tailor-made follow-up clinical protocols for primary prevention and screening in all immunosuppressed patients, based on the cause and severity of the immunosuppression [20].

From the analysis of the articles consulted, it can be understood that there is an increased risk presented in some of the reports may be due to the dual aspect of increased tumor-promoting inflammation due to active under-lying rheumatic disease and decreased anti-tumor cell mediated immunity due to treatment with immunosuppressive regiments. It is evident that several rheumatic diseases display elevated serum levels of inflammatory cytokines, such as IL-2, IL-6, TNF-α and markers of systemic inflammation. Furthermore, many of the major treatments discussed seem to affect cell-mediated immunity—they either downregulate expression of NKCs, CD8^+^ CTL cells, or thwart the Th1/Th2 balance towards the more anti-inflammatory Th2 profile, which is less effective in suppressing tumors than the Th1 cell mediated profile. It is arguable that combination of chronic elevation of pro-tumor inflammation due to disease flares, and the suppression of anti-tumor inflammatory roles, such as cell mediated immunity and cytotoxicity, by immunosuppressive treatments, may work together to increase the risk of HNCs in patients being treated for systemic inflammatory disorders.

While we have collected a body of evidence that warrants further study on the topic, there are many limitations to this report. For example, we only queried the most common and well-understood inflammatory disorders and their treatments. However, other conditions also lead to chronic systemic inflammation (e.g., tuberculosis, sarcoidosis, bullous pemphigoid, pemphigus vulgaris and ankylosing spondylitis), albeit they may not all be treated with immunosuppressive regiments. Furthermore, we did not investigate every possible treatment regimen used, nor the immune effects of combinatory use of several drugs at once. However, it is commonplace for patients to receive multiple regiments simultaneously to better control disease.

Moreover, many immunological studies we report only show the effects of treatment on the immune system over the short term, endpoints being measured any time between 4 weeks to only 12 months of drug administration. However, many of the rheumatic patients who developed HNCs did so due to often refractory, longstanding disease of several years. It is entirely possible that the short-term immunomodulatory effects of an immunosuppressant are completely different from its stabilized long-term effects.

Other limits of this study include the lack of reports on incidence of HNCs in patients with rheumatic disease. This may be due to report bias, especially with regard to case reports. Lastly, another limitation in this report is the lack of definition of HNCs by other reports—wherein we excluded esophageal, thyroid and skin cancers of the head from our definition, this may not be universal.

## 5. Conclusions

While no conclusions can be made with certainty, it is alarming that several studies report elevated risk of various HNCs in patients with systemic inflammatory diseases undergoing treatment. We argue that, per the immunological studies presented and the case reports and reviews, this increased risk may be through the combination of chronic increase in pro-tumor inflammatory factors due to disease activity and suppressed anti-tumor cell mediated immunity and cytotoxicity in response to the immunosuppressive treatments of the diseases. Regardless of whatever pro-oncogenic pathophysiology at play, it is warranted for oral- and non-oral physicians to continue investigating this risk, and its legitimacy. Similar to other cancers, HNCs are significantly more likely to be cured when diagnosed early. However, delayed diagnosis of HNCs may necessitate extremely invasive treatment, very poor prognosis, quality of life and healthcare cost. Therefore, it is an apt direction of study to delineate the incidence and pathophysiology of HNCs in systemic inflammatory diseases, to not only earlier diagnose HNCs in susceptible patients but also move towards prevent these devastating cancers.

## Figures and Tables

**Table 1:** Summary of risk of head and neck cancer (HNC), standardized incidence ratio (SIR) values and cytokines, chemokines and other immune/inflammatory response molecules in relation to HNC of patients with Systemic Sclerosis (SSc), Systemic Lupus Erythematous (SLE), Rheumatic Arthritis (RA), Ulcerative Colitis (UC), Crohn’s disease (CD), Psoriasis and Dermatomyositis (DM). Upward arrows indicate increase, downward arrows indicate decrease of compounds.

Disease	Risk of HNCs	SIR	Related Cytokines
SSc	Oral cavity and pharyngeal	3.67	IL-1, 1α, 2, 2R, 4, 6, 8, 10, 17, 18, 23 (↑) IFN-γ (↓) TNF-α (↑) TGF-β (↑) CXCL4 (↑)) CRP, MCP1, MIP-1γ, VCAM-1 (↑) MDC (↓)
	Oropharyngeal	9.63	
	Tongue	25	
SLE	Oropharyngeal	1.54–2.02	IL-2, 2R (↑) IFN-α, γ (↑) TNF-α (↓) TGF-β (↓)
	HNCs	2.02–2.16	
	Nasopharyngeal	2.02–4.18	
	Thyroid	2.31	
	Laryngeal	4.19	
RA	Oropharyngeal	–	IL-15, 20 (↑) TNF-α (↑) GM-CSF (↑)
UC	None	–	IL-2, 6, 8 (↑) TNF-α (↑) GRO (CXCL-1) (↑) Eotaxin (↑)
CD	Oral	–	Il-5, 6, 7, 8, 13 (↑) IFN-γ CRP (↑)
	Neck cavity		
	Tongue		
Psoriasis	Oral	0.7–2.1	IL-6 (↑) TNF-α (↑) CRP, ICAM-1 (↑)
	Pharyngeal	1.3–4.1	
	Laryngeal	1.43–2.9	
	Oropharyngeal	2.8	
DM	Nasopharyngeal	–	IL-1, 2R, 6, 15, 17, 18 (↑) IFN-signature (↑) TNF-R1 (↑) TGF- β (↓)

Abbreviations: CCL—chemokine ligands; CRP—C-reactive protein; CXCL—Chemokine platelet-activating factor; EGF—Epidermal growth factor; GM-CSF—family granulocyte-macrophage colony-stimulating factor; GRO—growth-related oncogene; HGF—hepatocyte growth factor; ICAM-1—Intracellular adhesion molecule 1; IFN-α/γ—Interferon alpha/gamma; IL—Interleukin; MAPK-1—Mitogen-activated protein kinase-activator protein-1; MCP1—Monocyte chemoattractant protein 1; MDC—Macrophage derived chemokine; MMP—matrix metalloproteins; PGE2—Prostaglandin E2; TGF-α/β—Transforming growth factor alpha/beta; TNF-α—Tumor necrosis factor alpha; VCAM-1—Vascular cell adhesion molecule 1; VEGF—vascular endothelial growth factor.

## Data Availability

Not applicable.

## Abbreviations

AIDS	Acquired immunodeficiency syndrome
AZA	Azathioprine
CCL	chemokine ligands
CD	Crohn’s disease
CRP	C-reactive protein
CsA	Cyclosporine
CTLs	Cytolytic T lymphocytes
CXCL	Chemokine platelet activating factor
CYC	Cyclophosphamide
DM	Dermatomyositis
DMARD	Disease-modifying antirheumatic drugs
EBV	Epstein-Barr virus
EGF	Epidermal growth factor
ESR	Westerngren erythrocyte sedimentation rate
GCS	Glucocorticoids
GM-GSF	Granulocyte macrophage colony stimulating factor
GRO	growth-related oncogene
HCMV-IgC	Human cytomegalovirus-immunoglobulin-G
HGF	hepatocyte growth factor
HIV	Human immunodeficiency virus
HNC	Head and neck cancers
HPV	Human papillomavirus
IBD	Inflammatory bowel disease
ICAM-1	Intracellular adhesion molecule 1
IFN-α/γ	Interferon alpha/gamma
IL	Interleukin
IL-2R	Interleukin 2 receptors
MAPK-1	Mitogen-activated protein kinase-activator protein-1
MCP1	Monocyte chemoattractant protein 1
MDC	Macrophage derived chemokine
MIP-1γ	Macrophage inflammatory protein 1 gamma
MMP	matrix metalloproteins
MTX	Methotrexate
NF-κB	Nuclear factor kappa B
NKCs	Natural killer cells
NPC	Nasopharyngeal carcinoma
PGE2	Prostaglandin E2
PM	Polymyositis
PMN	Polymorphonuclear neutrophils
RA	Rheumatoid Arthritis
SCCs	Squamous cell carcinomas
SIR	Standardized incidence ratio
SLE	Systemic lupus erythematous
SMD	Standardized mean differences
SSc	Systemic sclerosis
TAA	Tumor-associated antigens
TGF-α/β	Transforming growth factor alpha/beta
TNF-α	Tumor necrosis factor alpha
TNFi	Tumor necrosis factor inhibitors
T-regs	Regulatory T-cells
UC	Ulcerative colitis
VCAM-1	Vascular cell adhesion molecule 1
VEGF	vascular endothelial growth factor
